# Genome-Wide Genomic Analysis and Evolutionary Insights into Bovine Coronavirus Strains in Southwest China

**DOI:** 10.3390/vetsci12010009

**Published:** 2024-12-29

**Authors:** Qingqing Li, Huili Bai, Yan Pan, Yuying Liao, Zhe Pei, Cuilan Wu, Chunxia Ma, Zhongwei Chen, Changting Li, Yu Gong, Jing Liu, Yangyan Yin, Ling Teng, Leping Wang, Ezhen Zhang, Tianchao Wei, Hao Peng

**Affiliations:** 1 College of Animal Science and Technology, Guangxi University, Nanning 530004, China; ddliqingqing@163.com (Q.L.); baihuili2020@163.com (H.B.); cuilanwu@163.com (C.W.); machunxia1213@163.com (C.M.); 2 Guangxi Key Laboratory of Veterinary Biotechnology, Guangxi Veterinary Research Institute, Nanning 530001, China; liaoyuying@126.com (Y.L.); chen_zhong-wei@163.com (Z.C.); lctyq0508@163.com (C.L.); yinyangyan@163.com (Y.Y.); m15208987486@163.com (L.T.); wangleping96@foxmail.com (L.W.); 3 Key Laboratory of China (Guangxi)-ASEAN Cross-Border Animal Disease Prevention and Control, Ministry of Agriculture and Rural Affairs of China, Nanning 530001, China; 4 College of Animal Science and Technology, Guangxi Agricultural Vocational and Technical University, Nanning 530007, China; panyan413@163.com; 5 School of Neuroscience, The City College of New York, New York, NY 10031, USA; topeizhe@hotmail.com; 6 Guizhou Animal Husbandry and Veterinary Research Institute, Guiyang 550005, China; yituo-28@163.com (Y.G.); 18886066178@163.com (J.L.); 7 Institute of Agricultural Products Processing, Guangxi Academy of Agricultural Sciences, Nanning 530007, China; zhang281@126.com

**Keywords:** bovine coronavirus, whole-genome sequencing, cross-species transmission, genetic evolution analysis

## Abstract

The global epidemic of bovine coronavirus (BCoV) has caused enormous economic losses. The characterisation and genetic composition of endemic strains in Southwest China remain elusive. This study aimed to fill this gap by isolating three BCoV strains from this region and sequencing their whole genomes. To elucidate the genetic evolution and characterisation of the prevalent strains, the results of BCoV sequences were compared in GenBank, with a focus on genetic evolution, mutation, and recombination patterns. The results showed close homology between strains NN190313 and NN230328, while strain NN221214 showed less similarity to these two strains but clustered with the French strain of the European branch. Intriguingly, NN190313 and NN230328 were grouped with goat-derived BCoV strains from Jiangsu Province in Eastern China in the Asian–American branch. In addition, recombination analyses revealed significant signals between NN230328 and either a Chinese goat-derived strain (XJCJ2301G) or a Shandong strain (ShX310). This study highlights the importance of monitoring cross-species transmission between cattle and goats, especially in the mountainous areas of Southwest China where mixed farming occurs, and thus, the monitoring of cross-species transmission between cattle and goats is important for preventing new public health challenges, providing important insights for research on cross-species transmission, early prevention, and control measures, with potential applications in vaccine development.

## Simple Summary

Cattle play a vital role in livestock farming. Viral diseases in cattle, even contagious ones, can cause huge economic losses to the farming industry. Bovine coronavirus is one of them. Due to the paucity of studies on bovine coronavirus in Southwest China, the characterisation and genetic composition of strains prevalent in this region remain elusive. This study aimed to fill this gap by isolating and sequencing the whole genome of three BCoV strains from diarrhoeic calves from several large farms in Southwest China. This study underscores the importance of monitoring cross-species transmission between cattle and goats, particularly in the mountainous regions of Southwestern China where mixed farming practices are common and where it will be important to pre-empt new public health challenges. The comprehensive genomic analysis of these BCoV strains not only fills a research gap but also provides critical insights for cross-species transmission studies, early prevention, and control measures, with potential applications in vaccine development.

## 1. Introduction

Coronaviruses are the main pathogens that can cause severe respiratory and gastrointestinal diseases in humans, poultry, and wildlife [1]. In 1972, the Mebus strain, a bovine coronavirus (BCoV), was discovered and isolated in the United States [2]. Since then, BCoV has been detected worldwide, causing significant economic losses to the livestock industry. This genus of β-coronavirus is characterised by a high mutation rate and the capacity for homologous recombination, which significantly increases its risk of crossing species barriers. BCoV has been reported to infect a wide range of animals, including goats, cows, horses, moose, water deer, white-tailed deer, antelope, giraffes, llamas, dogs, cats, and even humans [3,4,5,6]. In 1988, a coronavirus closely related to BCoV called human enteric coronavirus (HEC 4408) was isolated from the faeces of children with diarrhoea in Germany, demonstrating a close relationship to BcoV [7]. Previous studies have reported a 96% homology between the HCoV-OC43 and BCoV strains, suggesting that HCoV-OC43 may have evolved through recombination between BCoV and other strains, with their most recent common ancestor dating back to around 1890. It is also hypothesised that the human coronavirus OC43 strain may have evolved through a recombination between BCoV and the porcine haemagglutinating encephalomyelitis virus [8,9].

BCoV can cause diarrhoea in calves aged 1–4 weeks, whereas in adult cattle, it causes calf diarrhoea (CD) and winter dysentery (WD) [10]. Symptoms in calves are mainly watery faeces and dehydration, whereas adult cattle may show signs of bloody dysentery. Additionally, BCoV can cause respiratory disease in both calves and adult cattle, characterised by coughing and difficulty breathing [11,12]. The virus is present throughout the year, with a higher incidence during the colder seasons. BCoV is categorised into two types based on clinical signs: bovine enteric coronavirus (BECoV) and bovine respiratory coronavirus (BRCoV). Both types can be found in the respiratory and intestinal tracts of healthy cattle. Since it was first isolated from diarrhoeal calves in the United States in 1972, BCoV has been reported in Europe, the Americas, Oceania, Africa, and Asia [13,14,15,16,17]. In 1994, Yang Shenghua and colleagues first isolated BCoV from the hearts and livers of cattle that died suddenly of yellow cattle syndrome in Jilin, China [12]. Since then, the presence of BcoV has been confirmed in many Chinese provinces, including Heilongjiang, Inner Mongolia, Henan, Shandong, Xinjiang, and Qinghai. The effects of BCoV include increased mortality in affected calves and reduced productivity in dairy and beef cattle, resulting in significant economic losses to the Chinese livestock industry [1,18,19].

**Figure 1:**
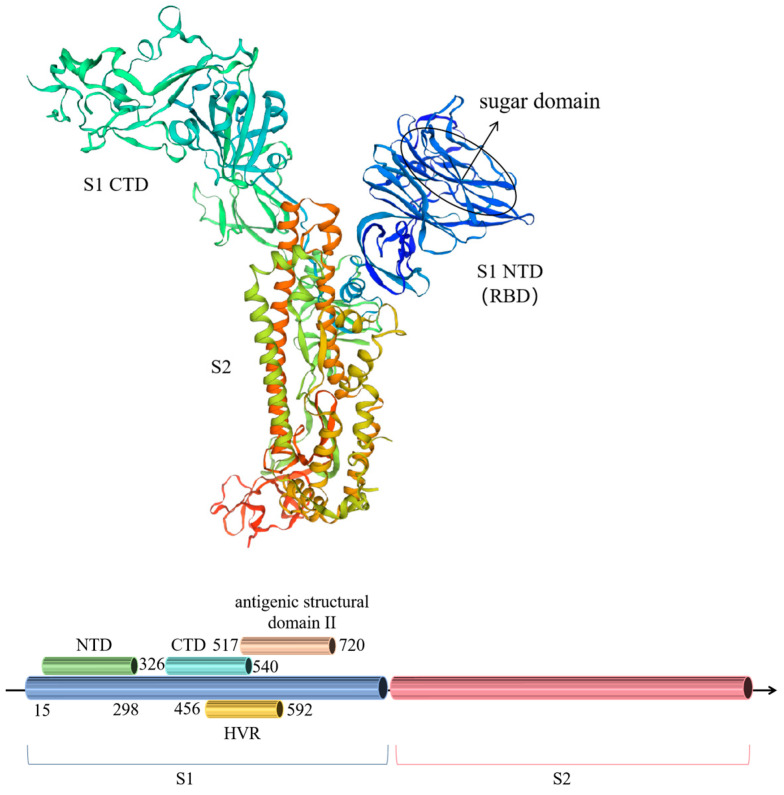
Crystal structure and functional domain distribution of the bovine coronavirus S gene.

The BCoV genome is characterised by a methylated cap structure at the 5′ end and a polyA tail at the 3′ end, along with two open reading frames (ORFs), ORF1a and ORF1b. It also includes functional genes for the hemagglutinin esterase protein (HE), the spike protein (S), the small envelope protein (E), the membrane protein (M), and the nucleocapsid protein (N). ORF1a and ORF1b constitute two-thirds of the genome, while the functional genes make up the remaining parts. Wu and Yan [20] highlighted that among coronavirus proteins, the spike (S) glycoproteins, which comprise the S1 and S2 subunits, show a higher susceptibility to mutations across different species. The interplay between the S1 and S2 subunits is critical in determining the virus’s affinity for host cells. Millet et al. [21] observed that the evolution of the coronavirus S gene, which includes the N-terminal domain (NTD) and C-terminal domain (CTD) binding modules, could enhance the adaptability of the S glycoprotein both within and across host species. Rekik and Dea [22] identified a highly polymorphic region (HVR) within amino acids 452 to 593 of the BCoV S gene, which is prone to mutation and recombination. Variations in this region might lead to changes in viral virulence, host range, and tissue specificity (Figure 1).

In this study, we focused on the whole-genome sequencing of three isolated bovine coronavirus strains. We conducted genetic evolution and recombination analyses of the entire genome and S genes to gain a better understanding of the characteristics and transmission patterns of Southwest China BCoV. Our findings not only support the study of human coronaviruses but also shed light on the cross-species transmission mechanisms of coronaviruses, offering insights for the prevention and control of bovine coronaviruses, which could present significant implications for public health.

## 2. Materials and Methods

### 2.1. Sample Processing and Cultivation of Cell Lines

During the period from 2019 to 2023, a total of 242 faecal, tissue, and nasal swab samples were collected from large-scale beef cattle farms located in Nanning, Chongzuo, Guigang, and Guilin, all of which are located within the Guangxi Zhuang Autonomous Region, China. These cattle were of all ages and were routinely immunised. The afflicted cattle exhibited varying degrees of diarrhoea; the faeces were notably foul-smelling, watery, and soft. In more severe cases, the presence of blood in the stool was observed, which occasionally led to death. After collection, all samples were immediately cooled for transport and subsequently stored at −20 °C. The HRT-18 cells used in this experiment were maintained by this laboratory.

### 2.2. RNA Extraction

For analysis, the samples were diluted at a 1:5 ratio. Viral RNA was extracted from the diarrhoeal faeces using the Viral Genome DNA/RNA Extraction Kit purchased from Kangwei Century Biotechnology Co., Ltd., Nanning, China, adhering to the manufacturer’s guidelines.

### 2.3. Reverse Transcription and RT-PCR

The RNA extracted from the samples underwent reverse transcription in a 20 μL reaction system. This system included 4 μL of 5× RT buffer, 4 μL of dNTP Mix, 2 μL of DTT, 2 μL of Primer Mix, 2 μL of RNase-Free Water, 5 μL of RNA template, and 1 μL of HiFiScript. The incubation process involved two steps: first, at 42 °C for 45 min, followed by at 85 °C for 5 min.

### 2.4. Cloning

RT-PCR products identified as positive were subjected to gel extraction using a commercialised Gel Extraction Kit (Omega, Norcross, GA, USA), following the manufacturer’s guidelines. The extracted DNA was then ligated into the pMD™18-T Vector (TaKaRa, Osaka, Japan) and transformed into DH5α competent cells (Sangon Biotech, Shanghai, China). The constructs were sent to Sangon Biotech (Shanghai, China) for sequencing to confirm the successful insertion of the target fragment into the pMD18-T Vector. Sequencing results confirmed the presence of BCoV gene sequences through BLAST analysis on the NCBI database.

### 2.5. Virus Isolation and Amplification

A total of 0.5–1 g of faecal samples was mixed with a PBS buffer at a 1:5 dilution ratio, vortexed, and centrifuged at 4 °C with 12,000 rpm for 10 min. The supernatant was collected, filtered through a 0.22 μm filter, and stored at −80 °C in an ultralow-temperature freezer. Initially, HRT-18 cells were washed three times with the PBS buffer. Samples that tested positive for the virus were then inoculated onto the HRT-18 cells. Trypsin was added to achieve a final concentration of 2 μg/mL, and the cells were incubated at 37 °C for 1.5 h with gentle shaking every 15 min. After this, the viral medium was discarded, and the cells were washed 2–3 times with PBS. The serum-free 1640 medium containing trypsin at a final concentration of 1 μg/mL was then added. The viral solution was collected and stored at −20 °C once over 50% of the cells had detached. Following three passages of blind passaging using the described methods, RT-PCR was performed using the primers detailed in Section 2.3. Subsequent cloning and sequencing procedures were conducted as outlined in the previous section.

There were 44 pairs of primers (see Table 1) designed by our lab to detect the target fragments. The cloned samples were sent to Sangong Bioengineering Co., Ltd. (Shanghai, China) for sequencing, and the sequencing results were compared and verified by BLAST in the NCBI database. The whole genome of the virus was sequenced three parts at a time, and finally, all the results were spliced together to obtain the whole genome of the virus.

### 2.6. Sequence Assembly and Genetic Evolution Analysis

The sequences of the three strains were assembled using SeqMan in DNASTAR 7.1 software sourced from Kerida (Beijing) Technology Co. The amino acid sequences of the functional genes investigated in this study were deduced using BioEdit within DNASTAR software. The nucleotide sequences of the three isolates were aligned with those of α, β, γ, and δ coronaviruses, BCoV, and bovine-like coronaviruses, as well as with reference sequences of the S functional genes obtained from GenBank. These sequences were subjected to multiple-sequence alignment using Clustal W 2.1 software. MegAlign in DNASTAR software facilitated the analysis of nucleotide and amino acid sequence similarities. The most appropriate model for phylogenetic analysis was selected using jModelTest 2.1.7 software downloaded from https://jmeter.apache.org/, (accessed on 5 October 2023) and the phylogenetic tree was constructed using the maximum likelihood method via the IQ-TREE web server (http://iqtree.cibiv.univie.ac.at/ (accessed on 5 October 2023) with 1000 bootstrap replicates.

### 2.7. Recombination Analysis

A recombination analysis of the S genes from the three isolates was conducted using two different software programs designed for recombination detection. Within RDP 2.2 software, seven distinct algorithms (RDP, GENECONV, Bootscan, MaxChi, Chimera, SiScan, and 3Seq) were employed to assess the S genes for evidence of recombination. The presence of recombination breakpoints was confirmed when at least four of these methods identified recombination breakpoints, and a *p*-value of less than 10^−6^ indicated that the detection results were reliable. Recombinants displaying significant recombination signals within RDP software were further validated using the Simplot recombination analysis tool. Consistency between the results obtained from these two analytical methods confirmed the credibility of the detected recombination signals.

### 2.8. Selection Pressure Analysis

To investigate the evolutionary pressures influencing BCoV evolution, we estimated the ratio of non-synonymous (dN) to synonymous (dS) variations per site using a codon-based maximum likelihood method. A dN/dS ratio greater than 1 indicates positive or diversifying selection, a ratio less than 1 suggests negative or purifying selection, and a ratio equal to 1 signifies neutral evolution. The selection pressure on the positive selection sites within the BCoV S gene was analysed using four methods—Fixed-effects Likelihood (FEL), the Mixed-effects Evolutionary Model (MEME), Single-Likelihood Ancestor Counting (SLAC), and Fast Unconstrained Bayesian AppRoximation (FUBAR)—via the online tool Datamonkey (www.datamonkey.org). These methods consistently identified loci as positively selected, confirming their significance. To enhance the accuracy of and minimise false positives in the selection pressure analysis, the presence of recombination was evaluated using the Genetic Algorithm for Recombination Detection (GARD).

## 3. Results

### 3.1. Genome-Wide Genetic Evolutionary Analysis

In this study, three strains of BCoV from Nanning, Guangxi, were isolated and named NN190313, NN221214, and NN230328. No viruses were isolated from other samples. The genome of NN190313 is 30,976 bp long, with an A + T content of 62.93% and a C + G content of 37.07%. NN221214 has a genome length of 30,975 bp, featuring an A + T content of 62.88% and a C + G content of 37.12%. The NN230328 genome spans 31,000 bp, with an A + T content of 62.97% and a C + G content of 37.03%.

**Figure 2:**
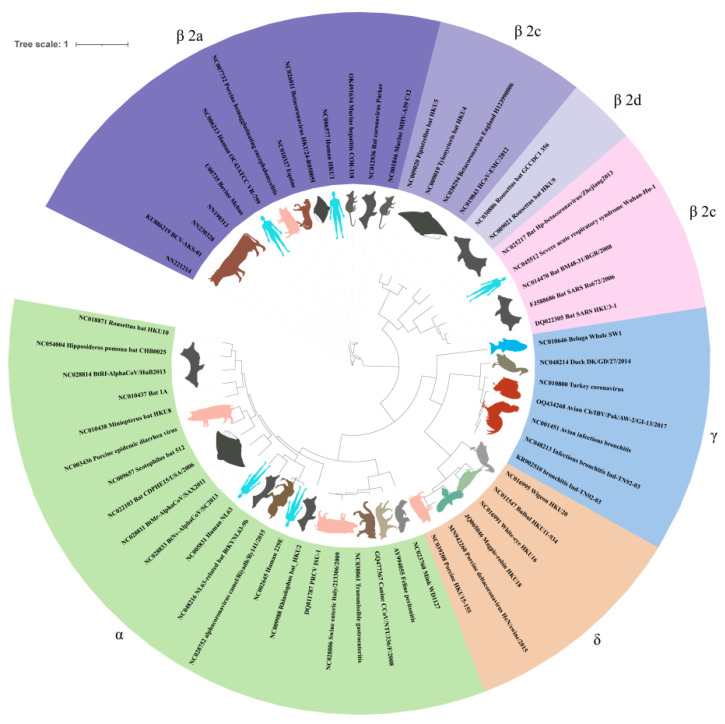
Genome-wide genetic evolutionary tree of 3 isolates of BCoV and 57 strains of α, β, γ and δ coronaviruses (ML method).

The complete genome sequences of these three BCoV strains were compared for homology with reference sequences from 57 strains of α, β, γ, and δ coronaviruses. This analysis revealed that BCoV exhibited the highest homology with other BCoV strains, with similarities ranging from 97.3% to 98.3%. They also showed significant homology with human coronavirus OC43, with percentages ranging from 94.7% to 95.7%, and with the porcine hemagglutinating encephalomyelitis virus, showing similarities between 91.7% and 92.4% (Table 2). The optimal model for analysing the whole-genome sequences of these three bovine coronavirus strains and the reference sequences from the 57 strains of α, β, γ, and δ coronaviruses was determined to be GTR + G + I. The genetic evolutionary analysis was conducted using IQ-TREE to construct a maximum likelihood (ML) phylogenetic tree, as illustrated in Figure 2. The phylogenetic tree displays four main branches, with the three β-coronavirus isolates further subdivided into four sub-branches: a, b, c, and d. The three bovine coronavirus strains, along with the members of the β-coronavirus subgroup a, cluster together to form a larger branch.

**Figure 3:**
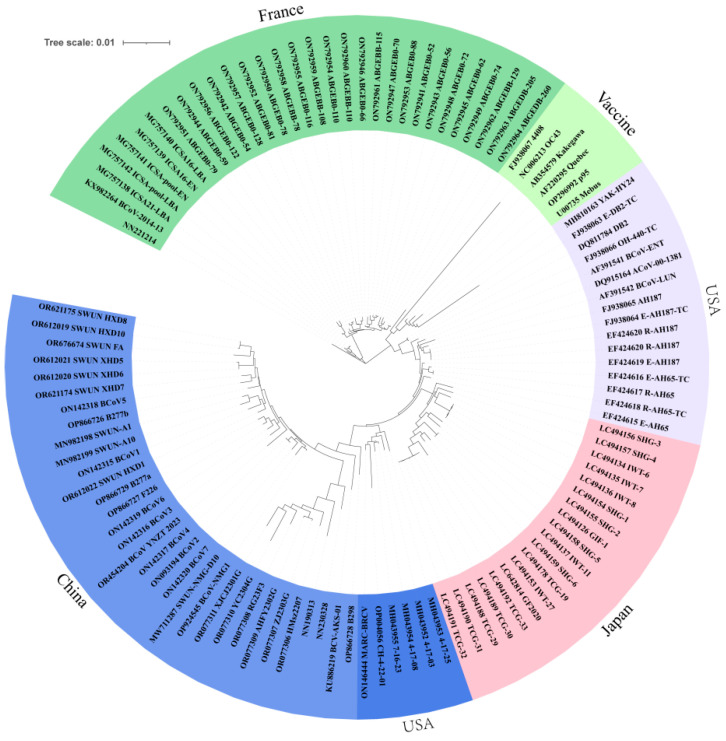
Genome-wide genetic evolutionary tree of 3 isolates of BCoV and 117 strains of BCoV (ML method).

Homology analysis revealed high similarity among the strains: 95.2% between NN190313 and NN221214, 96% between NN190313 and NN230328, and 97.7% between NN221214 and NN230328. These isolates also exhibited 95–98.9% nucleotide homology with other coronaviruses, including 97.5–97.9% with the vaccine strain Mebus and 94.8–95% with human coronavirus OC43 (Table 2). Notably, strain NN221214 showed the highest homology, at 98.7%, with the French strains ICSA-pool-EN, ICSA16-LBA, ICSA16-EN, and ICSA21-LBA. Similarly, NN190313 exhibited the highest similarity, also at 98.7%, with bovine coronavirus strains of goat origin from Jiangsu, China, including HMsz2207, AHFY2302G, XJCJ2301G, and ZJ2303G. Meanwhile, the NN230328 strain shared the highest homology, at 98.9%, with strains B277a/2021 from Shaanxi, China, and BCoV2/2021/CHN from Heilongjiang, China. The complete genome sequences of the 3 bovine coronavirus strains and the reference sequences of 117 bovine coronaviruses were best modelled using the GTR + G + I model. The maximum likelihood phylogenetic tree, constructed using IQ-TREE, revealed a distinct geographical distribution in its branching (Figure 3). European strains formed one cluster, Asian–American strains formed another cluster, and vaccine strains were grouped individually. The three isolates from this study were situated on different evolutionary branches: NN221214 clustered primarily with European strains from countries such as Sweden, France, Ireland, Denmark, and Italy, specifically forming a smaller branch with the French strain ICSA21-LBA. Both NN190313 and NN230328 were grouped together with the bovine-like coronavirus XJCJ2301G and CH-4-22-01.

### 3.2. S Gene Analysis

**Figure 4:**
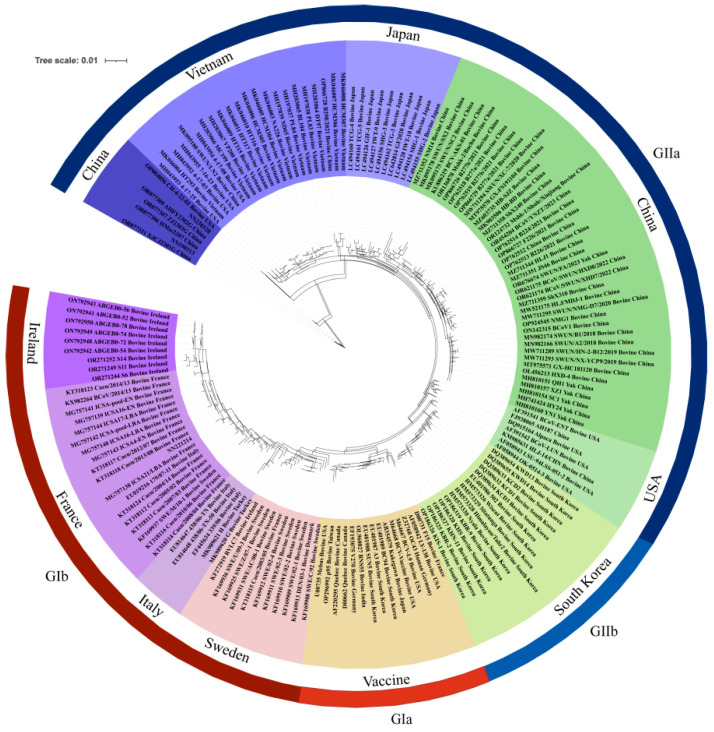
Genetic evolutionary tree of S genes of 3 isolates of BCoV and 159 strains of BCoV (ML method).

The S gene of the three isolates measured 4092 bp in length, encoding a total of 1364 amino acid (aa) residues. An analysis of the S gene revealed mutations; however, no shifts, deletions, insertions, or nonsense mutations were observed. A comparison of the nucleotide sequences of these three strains with 159 BCoV S genes from the NCBI database demonstrated homology levels ranging from 94.3% to 96.9% with the vaccine strain Mebus, 93.4% to 95.5% with the American elk-origin BCoV strain CH-4-22-01, and similarly, 93.4% to 95.5% with human coronavirus OC43 (refer to Table 3). The TN93 + G model was identified as the optimal model for analysing the S gene, and a maximum likelihood (ML) phylogenetic tree was constructed using IQ-TREE (Figure 4). This evolutionary tree exhibited a typical geographical distribution, dividing into two principal branches based on genotype: the European strain genotype I (GI) and the Asian–American strain genotype II (GII). The branch including the Mebus vaccine strain was further subdivided into GIa, while the European strain was classified under the GIb subgroup. Meanwhile, the branch associated with Korea fell under the GIIa subgroup, and the branch encompassing China, the United States, Japan, and Vietnam was designated as GIIb.

In this phylogenetic analysis, strain NN221214 was clustered with the French strains ICSA21-LBA, Caen/2013/08, and Caen/2012/07, categorising it within the GIb subgroup. Conversely, strains NN190313 and NN230328 were grouped with four goat-origin BCoV strains from Jiangsu Province, China, indicating their affiliation with the GIb subgroup. Additionally, these two isolates, NN190313 and NN230328, along with four goat-derived BCoV strains from China (HMsz2207, AHFY2302G, ZJ2303G, and XJCJ2301G) and an elk-derived BCoV strain CH-4-22-01 from the United States, formed a distinct branch within the GIIb subgroup.

The comparison of the S gene amino acid (aa) sequences among three BCoV strains with multiple sequences of the BCoV strain highlighted significant variability. Specifically, strain NN190313 exhibited considerable divergence, presenting a total of 65 amino acid variants. Among these, 49 were identical to those found in goat-origin BCoV strains, while 16 were unique to NN190313, including L242P, T257S, T422I, S458A, I503V, P550T, R597G, N676S, F693I, Y774H, N810D, D825H, A1119T, N1149K, Y1155D, and F1339V. Strain NN230328 exhibited 30 amino acid variants, with 21 matching those of the XJCJ2301G strain and 13 aligning with the variants of the Shandong, China, ShX310 strain. This strain also had eight unique variants: N178T, V467A, T856I, N908Y, G1026D, E1082G, G1093V, and D1248N. Lastly, strain NN221214 revealed a total of 22 amino acid variants, with 20 being identical to those of the ICSA21-LBA strain and 1 unique variant, T540I (Table 4).

### 3.3. S1 Subunit HVR

**Figure 5:**
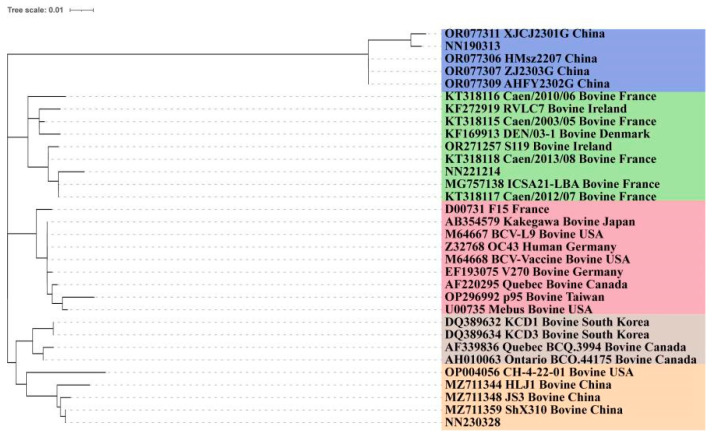
Genetic evolution tree (ML method) of the HVR (S gene 452-593 nt) of 3 isolates of BCoV.

A multiple-sequence alignment of the nucleotide sequences within the S1 subunit Highly Variable Region (HVR) of the S gene demonstrated that the nucleotide sequences of the three strains were homologous to other BCoVs, as illustrated in Table 5. Homology to the vaccine strain Mebus ranged from 85.3% to 95.7%, and to human coronavirus OC43, it varied from 85.7% to 96.5% (refer to Table 5). Strain NN221214 exhibited the highest homology with the French strains ICSA21-LBA and Caen/2012/07. Strain NN190313 demonstrated the highest homology with the Chinese goat-origin BCoV strain XJCJ2301G. Strain NN230328 exhibited 100% homology with the Chinese Shandong strain ShX310. In phylogenetic clustering, NN221214 was grouped with the French strains, NN190313 with the Chinese goat-origin BCoV strain, and NN230328 with strains from Shandong, Sichuan, and Heilongjiang in China (see Figure 5).

In the amino acid sequence comparison of the HVR, NN221214 shared identical amino acid variants with the French ICSA21-LBA strain. The strain NN190313 had 19 amino acid variants in total, with 16 matching those of the XJCJ2301G strain and 3 unique variants, as detailed in Table 4. Similarly, the NN230328 strain and the ShX310 strain shared exactly the same amino acid variants.

### 3.4. Results of Recombination Analysis

**Figure 6:**
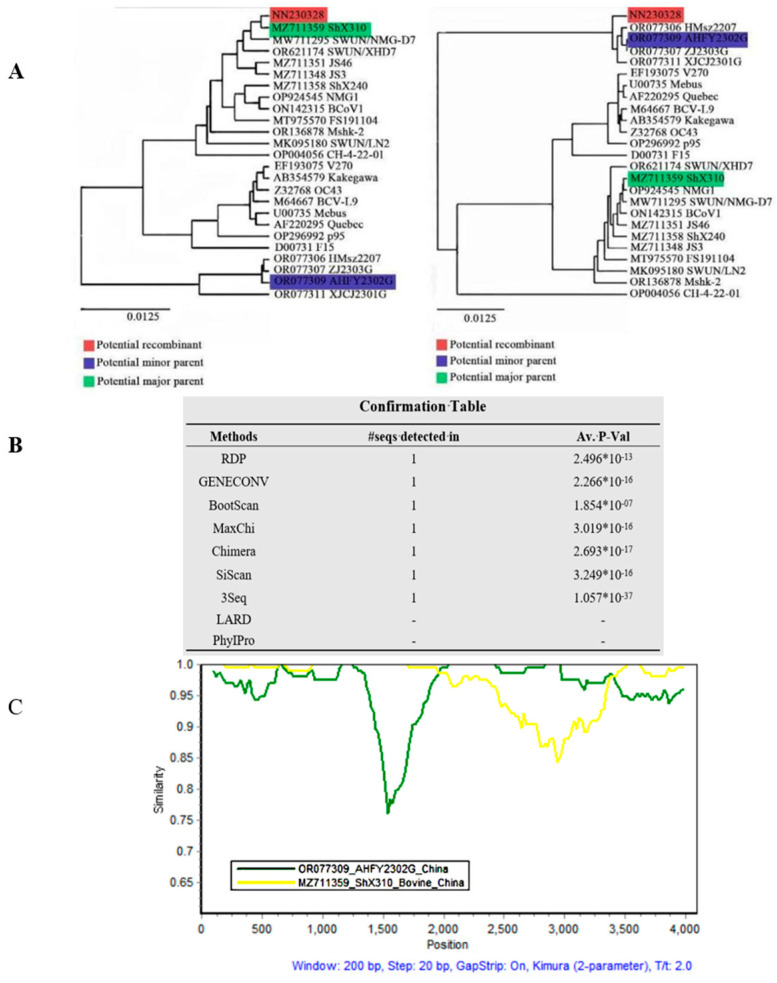
Recombination analysis of S genes of three strains of bovine coronavirus in RDP4.0 and Simplot software ((**A**) is the ML genetic evolution tree built by RDP4.0; (**B**) is the algorithmic *p*-value in 7; (**C**) is the locus map of recombination analysis by Simplot software).

A recombination analysis of the S genes from the three strains was conducted using two different types of recombination software, yielding consistent results across both platforms (see Figure 6). The S gene of strain NN230328 was detected as a significant recombination event using seven algorithms of RDP software: RDP, GENECONV, Bootscan, MaxChi, Chimera, Siscan, and 3Seq (*p* < 10^−6^). This analysis suggested that the primary parent of this recombination event was the strain ShX310 from Shandong, China, and the secondary parent was the goat-derived coronavirus strain AHFY2302G from Jiangsu, China (Figure 6). Strain NN230328 exhibited 99.8% similarity to the primary parent and 99.6% similarity to the secondary parent. Recombination breakpoints with ShX310 as the main stem were identified in the ranges of 1–1969 and 3436–4095, while segments 1970–3435 were influenced by AHFY2302G. Additionally, Simplot 3.5.1 software detected that 1961–3381 were mutated by AHFY2302G. Although the recombination breakpoints seen by RDP and Simplot software were different, the regions where recombination was detected remained consistent between both analyses.

### 3.5. Selection Pressure Analysis

Selection pressure analysis was performed by testing 142 strains of the bovine coronavirus S gene; codon 11 was detected as a positive selection site (Table 6), position 12, 113, 501, and 509 were detected as positive by four methods, and position 447, 458, 499, 510, 770, 1238, and 1363 were detected as positive by three methods.

## 4. Discussion

The whole genomes of three BCoV isolates from this study were used to establish an evolutionary tree with four genera of coronaviruses, α, β, γ, and δ. All three recently isolated BCoVs were clustered in the same branch with other BCoV strains and compared with the other coronaviruses. The results demonstrated that human coronavirus OC43 had the highest homology with BCoV, which ranged from 94.7% to 95.7%, followed by the homology of porcine hemagglutinating encephalomyelitis virus. A multiple sequence comparison of the S genes showed that the homology of BCoV and OC43 was 94.3–97%. It is worth noting that OC43 and porcine hemagglutinating encephalomyelitis virus belong to the same genus and subgroup. This BCoV and HCoV-OC43 as well as other BCoVs also share the same cell-binding receptor, 9-O-acetyl-sialic acid (9-O-Ac-Sia), which facilitates cross-species transmission, especially to humans, suggesting the need for further analysis and a closer monitoring of any further changes to these BCoV strains.

The BCoV S gene is most prone to mutation, especially in the S1 subunit region, which plays an important role in the cross-species transmission of BcoV [23]. The functional S protein mainly mediates adsorption, membrane fusion, and hemagglutination between the virus and the target cell receptor [24]. Particularly, its S1 subunit functions in receptor recognition and induces the production of neutralising antibodies, which are prone to mutation, leading to changes in the antigenicity of the virus and the viral pathogenicity of the virus [25]. Meanwhile, the S2 subunit functions as a mediator for the fusion of the virus and the cell membrane, and the S1 subunit and S2 subunit are both critical in determining the host range and the histophilicity of the host [26]. The NTD region of the S1 subunit of BCoV is located at amino acids 15–298 and the CTD region is located at amino acids 326–540 of the S gene [27]. The structural NTD and CTD have the potential to bind to different types of receptors, and the NTD binds mainly to carbohydrate receptors such as polysaccharides and glycoproteins, and the main binding partners of the CTD are protein receptors. Both NTD and CTD can act as receptor-binding structural domains (RBDs) that bind to receptor salivary acid, thus helping to determine the host range of coronaviruses and cross-species infections [28]. BCoV uses the NTD as an RBD, and the NTD may play a key role in species barrier-crossing events that allow emerging coronaviruses to adapt to diverse new host environments by binding to the receptor via the salivary acid in the host cell. Mutations in structural CTDs cause the virus to adjust and acquire adaptive mutations to optimise binding to the new host protein receptor. Dongwan Yoo [28] found that the S1 subunit of the S gene contains two important neutralising epitopes, A and B. The antigenic structural domain is located between amino acids 324 and 403 of I and the antigenic structural domain II is located between amino acids 517 and 720, both of which are associated with the induction of neutralising antibodies, and the two antigenic determinants may be related to polymorphisms in the S1 subunit.

Based on their geographic distributions, BCoVs were categorised into two genotypes, GI and GII, with the GI genotype being the European strain and the GII genotype being the Asian–American strain. The distribution of the evolutionary tree had the same geographical distribution as that of the whole-genotype strains, with the European strains represented by Ireland, France, and Italy clustered together, the Asian–American strains dominated by China, the United States, Japan, Korea, and Vietnam clustered together, and the vaccine strains clustered in a separate branch. The strain NN221214 was clustered with the French strains ICSA21-LBA and Caen/2012/07 in the European branch. Amino acid mapping of the S gene revealed that NN221214 shared 22 amino acid variants with the French strains, of which 13 occurred in the S1 subunit region and 9 in the S2 subunit region. This may be because China introduced good-quality cattle from France to breed with local cattle 20 years ago. Charolais cattle from France are famous for their cold and heat resistance, fast growth, and good meat quality, while Limousin cattle have strong adaptability and high meat production performance, and these two breeds are more widely bred in the south of China. Recently, the crossing of Limousin cattle with local yellow cattle for breed improvement has become popular among local farms in the Guangxi Region of China. The NN221214 strain is genetically close to the French strain, whereas the spread of this strain may be related to China’s introduction, transportation, and trading either globally or domestically.

A genetic evolutionary analysis of the whole genome and S gene of both strains, NN190313 and NN230328, revealed that they clustered into a branch with the Chinese goat-origin BCoV strain, which belonged to the sub-American branch. An amino acid sequence alignment of the S genes showed that the strain NN190313 shared 49 identical amino acid variant sites with the goat-origin BCoV strain XJCJ2301G, with 27 occurring in the S1 subunit region and 22 in the S2 subunit region. Notably, 35 amino acid variation sites were present only in BCoV and bovine-like coronaviruses (Chinese goat-origin XJCJ2301G and American elk-origin CH-4-22-01), of which 21 were unique to the isolated goat-origin coronaviruses, and 14 were common amino acid variation sites between the isolated goat-origin coronaviruses and the American elk-origin BCoV strain CH-4-22-01.

The NN230328 strain shared 18 amino acid variation sites with the goat-derived BCoV strain XJCJ2301G, with 5 occurring in the S1 subunit region and 13 in the S2 subunit region. Among them, four sites were unique to the isolated goat-derived coronaviruses, and nine were amino acid variation sites shared by the isolated goat-derived and elk-derived BCoV strain CH-4-22-01. The NN230328 strain was associated with Chinese strains BCoV/SWUN/XHD7, SWUN/NMG-D7, ShX310, and HLJ/MDJ-1 from Shandong, Sichuan, and Heilongjiang. 174, 501, 509, 510, 718, 769, and 9277 loci manifested as polymorphic mutations. Amino acids 146, 148, and 154 of NN190313 and NN230328 were located near the sialic acid binding site in the structural NTD of the S1 gene, and mutations in these amino acids may affect the virus’s binding to the cellular receptor and lead to cross-species transmission. Changes in amino acid sites 146, 148, 501, and 509 (amino acid sites 146, 148, and 509 gradually converted to 146 N-146 I, 148 D-148 G, and 509 N-509 T, respectively, over time) were found to result in structural changes in the protein. Previous studies have found that the BCoV strain in the United States also undergoes mutations at three amino acid sites and hypothesised that mutations at these sites may be important for distinguishing between the GI and GII groups [25]. When the SWISS-MODEL-predicted crystal structures of S1 proteins of a group of strains (NN190313 and NN230328 isolates, bovine-like coronavirus, vaccine strain Mebus, and HCoV-OC43) were compared, it was found that variation in the 510 amino acid caused changes in the secondary and tertiary structures of BCoV, bovine-like coronavirus, and HCoV-OC43 proteins at 508–511, and the two isolates at positions 501, 509, 510, 525, 527, 528, and 531 caused changes in the secondary and tertiary structures of bovine coronavirus and bovine-like coronavirus.

The HVR is prone to mutation and recombination, leading to changes in virulence, strain host range, and tissue tropism [21,22]. A further analysis of the HVR of the S gene revealed that the NN190313 strain clustered in a single branch with Chinese goat-derived bovine coronavirus strains alone. It had the highest homology and 16 common amino acid variants with the Chinese goat-derived bovine coronavirus strain XJCJ2301G. Additionally, the NN230328 strain was clustered in a single branch with Chinese strains from Shandong, Sichuan, and Heilongjiang, sharing 100% homology and exactly the same amino acid variants with the Chinese Shandong strain ShX310. Based on the analysis of strain NN230328’s S gene and its HVR, we hypothesised that recombination might have occurred. By analysing the S gene of the three bovine coronavirus strains, we found that the S gene of the NN230328 strain exhibited significant recombination signals with the goat-origin bovine coronavirus strain XJCJ2301G from Jiangsu, China, and also the bovine coronavirus strain ShX310 from Shandong, China. The recombination fragments occurred between the S1 and S2 subunits, primarily in the S2 subunit region.

It has been found that single-point mutations in the antigen domain lead to a loss of neutralising activity. Furthermore, single-point mutations within S protein neutralising epitopes are generally responsible for the evasion of BCoV from immunoselective pressure. 

The NN190313 strain, as well as bovine and sheep coronaviruses, has 3 unique amino acid mutations in antigen domain II, while goat-derived bovine coronaviruses have 9 unique amino acid mutation check points (535, 542, 543, 558, 568, 583, 604, 622, 720). Immune escape may be the natural evolutionary mode of exposure to the host immune system, and a selection pressure analysis of the bovine coronavirus S gene revealed 11 codons under positive selection pressure at positions 12, 113, 447, 458, 499, 501, 509, 550, 770, 1238, and 1363. In conclusion, our analysis suggests that the S genes, specifically the S1 subunit, are likely driven by the changes in amino acids that occurred through geographic, environmental, and natural selection patterns that have mutated the amino acids for greater environmental adaptation. It was found that most of the unique variant sites of NN190313 and NN230328 and the recombination phenomenon detected in this study occurred in the S1 subunit. Therefore, we speculate that the unique amino acid variants and recombination found in BCoV and bovine-like coronavirus strain S1 subunits in this study may cause cross-species transmission between cattle and goats by altering neutralising antibody recognition, cellular receptor structure, immune evasion, or the host range and viral adaptation.

Strains NN190313 and NN230328 were isolated and preserved by our laboratory in Guangxi, China, in 2019 and 2023, respectively. A time projection of the isolation of NN190313 and NN230328 indicated that goat-derived strains of bovine coronavirus (BCoV) have been prevalent in the southern region of China in the last three years, which provided the possibility of virus recombination and the emergence of new strains. The NN190313 strain isolated in this experiment can be transmitted between cattle and goats, which may be related to the mode of free-range farming in the mountainous areas of Southwestern China and the farming environment, leading to the cross-species transmission of BCoV, which has been shown to have the ability to infect a wide range of wildlife and humans, and SARS-CoV, which is a genus of β-coronaviruses, also belongs to the same genus as BCoV and spreads through many intermediate hosts to humans, such as civets, bats, and so on. HCoV-OC43 may have evolved from BCoV strains. These results suggest that β-coronaviruses may undergo repeated interspecies transmission. Notably, these β-coronaviruses may have the same intermediate hosts and thus have important implications for cross-species transmission in cattle. This also suggests that mutation in and the recombination of BCoV may lead to changes in viral immune evasion and host range, which further increase the difficulty of prevention and control. Therefore, it is even more important to focus on the prevention and control of BCoV in order to avoid biosecurity problems and serious economic losses.

In this study, we analysed the full gene sequences of three isolated strains to gain a deeper understanding of the prevalence of bovine coronaviruses in Southwest China. The findings of this study provide an important reference for prevention and control strategies. The analysis of the S proteins of BCoV and their amino acid variations provides data to understand the genetic characteristics of prevalent strains, viral variation, host adaptation, and cross-species transmission. Consequently, studying the S gene holds significant importance for virus research and vaccine development. This study also lays the foundation for subsequent research steps. In addition, the impact of these amino acid variations on viruses and viral protein functions warrants further investigations, which is an integral part of our forthcoming work.

## 5. Conclusions

Herein, we isolated three strains of BCoV, and two goat-origin bovine coronaviruses were detected through the three strains of BCoV. We speculate that goat-derived bovine coronavirus strains may be prevalent in Guangxi or even in the Southwest region and may be transmitted between cattle and goats, which may be related to the introduction of strains from different regions of the country and the breeding pattern in the mountainous region of Southwest China. BCoV and BCoV-like strains have multiple identical amino acid mutations, and the mutations lead to changes in the secondary structure of the proteins, which may play an important role in the cross-species transmission of BCoV. Amino acid mutations in the lectin and esterase domains of the HE gene of the three isolates may lead to changes in the receptor-binding domains, and the HE gene and the S gene together affect the binding of the virus to the cellular receptor and the spread of BCoV across species. This study increases the research data for BCoV in the Guangxi Region and provides a theoretical basis for the prevention and control of BCoV in the Guangxi Region.

## Figures and Tables

**Table 1:** Primer pairs for whole-genome amplification of BCoV.

Name	Length	Nucleotide Sequence (5′-3′)	Name	Length	Nucleotide Sequence (5′-3′)
1F	1672	TCCCGCTTCACTGATCTCTT	19F	1181	AGTGCCTTTCAACAGGTATT
1R		GCCTTGCAACCTACAACTCC	19R		TACTATAATTCTCCTGCGGC
2F	1211	CTAGCACAATCTTCAGGTGT	20F	1369	CATACTGGCTAATGAATGTA
2R		CAACCACAACAGGGTAATAT	20R		GCTTCAAACAACTGCATATT
3F	1228	TTCATCTCAATTGCCATTAG	21F	1133	GAAGGCTAACCCTTTGTGGC
3R		ACACACTGCCAATCACATAA	21R		GGCCAAACACCGTGTCATTA
4F	1243	GCCAGTGAACAGGAGGAGTC	22F	1129	TGGCGCCATAGTGTTACATG
4R		TCTCGGCAGTACCAAGTAGA	22R		ATCCCAAATGACGTGCTTCC
5F	987	GGTGGTGTTGCAAAGGCTAT	23F	617	CACGAGAGTGTTGAATAATT
5R		TAGGCAAGCAATTCCTTCTG	23R		ATCCCAAATGACGTG.CTTCC
6F	1211	AATAAGGCTACTGTTGCTCA	24F	1103	GGCTAAGATCGATAAGGAGG
6R		CACCTGTAGCTGTTGGCCAC	24R		CTTCCCATAATTCCAGAGAC
7F	1135	CCTGACTTGTCACAATATTA	25F	982	TGCTTTGGTGTAACGATGAG
7R		ACATGCGATACTTCCATTAC	25R		GAATATCCAGCATAGCAAGA
8F	1220	GGAGTGTGGTTGCTAGAGGT	26F	1095	ACAAGCCTAATCACTTTATC
8R		GAAGAATGCGCAGTTGCTAT	26R		GTTGACCTTCGCCTGTGTAA
9F	1162	TGGTAGTGGAAAATGATGCC	27F	1324	AAGAAGACTAACCTCAGTGTAAATG
9R		GCCCCTCTGTATAACAATAA	27R		CATCTTTAGATTATGGTCTAACCAT
10F	1367	GATGGAGTGCAGTGTTATAC	28F	1122	ATGTTTTTGATACTTTTAATTTCCT
10R		GGCGTACGAGAATTTTGCAG	28R		ACAAGTAAATGAGTCTGCCTGAATA
11F	1032	TTACCCTACACTTTCTTGTG	29F	1396	TATTCAGGCAGACTCATTTACTTGT
11R		GTACCAGTATGACAACCAGT	29R		ACAGACAAAAGCAGAACAATCAATA
12F	988	TGAATGGTTTATGGTTGGATGA	30F	1583	CCTTCAGAGTTTACTATAGG
12R		TAACCAACAACATGGCCAAA	30R		AGTCCCACATCCTGTACAAC
13F	1104	CAACAACTCGCTGGTATCAA	31F	1616	TGTGGTGATTATGCAGCATGT
13R		CGCAAACTTCTTCAAT ACTA	31R		ATATAAGACACAGTACCCAC
14F	1156	TTGTGGCAGTATTGTAGCAC	32F	1519	CT AC ATC AATCTC AAG GACA
14R		AGGATCTACAGAAAACGCAC	32R		CTGGGTTGAAACTCCACCAA
15F	1100	ACTGTTCAAGATGTTAAAGG	33F	623	ATAGGTATTGACACTAGCAC
15R		ACCTCCACCAATTTGTCTGC	33R		TGCCTACCTCTATTAAACAC
16F	1001	GGATCTTTGCTATGCGTTGC	34F	905	ACCTGGACTGCTGATGAAGC
16R		CAACTGCTTAATGTCCACCA	34R		CACCTTGTCCCTCTGCAAAT
17F	887	GTTGCGGCTATAACAAGCGG	35F	1440	GACACCGCATTGTTGAGAAA
17R		GCCACCAGGCTTAACATAAT	35R		GGTGCCGACATAAGGTTCAT
18F	835	TTCCTGTTGTTATAGGCACC	36F	935	TGATATGGCTGATCAAATTGC
18R		GCAAGACTTACAAATCGTTC	36R		CAGTGACCGTGATTCTTC CA

**Table 2:** Homology of 3 isolates of BCoV with α, β, γ and δ coronaviruses (between-genus) and 117 strains of BCoV (interspecies).

	Between-Genus			Interspecies		
	NN190313	NN221214	NN230328	NN190313	NN221214	NN230328
BCoV	97.3–98.3%			95–98.9%		
Mebus	-			97.5–97.9%		
OC43	94.7–95.7%			94.8–95%		

**Table 3:** Homology of S gene and HVR of 3 isolates of BcoV.

	S Gene			HVR		
	NN190313	NN230328	NN221214	NN190313	NN230328	NN221214
BCoV	93.2–98.9%	94.9–97.1%	93.6–99.4%	84.9–98.9%	85.1–100%	85.3–98.9%
Mebus	94.3%	96.9%	95.9%	85.3%	95.7%	94.4%
OC43	94.2%	96.1%	97%	85.7%	96.5%	95.2%

**Table 4:** Amino acid variant sites in the S gene of three BCoV identical to the reference strain.

NN190313				NN230328				NN221214	
XJCJ2301G/AHFY2302G				XJCJ2301G		ShX310		ICSA21-LBA	
aa Changes	S Gene Location	aa Changes	S Gene Location	aa Changes	S Gene Location	aa Changes	S Gene Location	aa Changes	S Gene Location
N-I	146	S-A	622	N-I	146	A-T	12	I-T	113
D-G	148	Q-E	720	D-G	148	E-V	121	D-N	115
L-F	154	S-A	769	L-F	154	H-R	143	H-Y	143
N-H	169	S-A	786	P-S	174	D-G	148	L-F	147
N-H	173	S-A	827	S-P	501	L-F	154	L-I	151
L-M	256	S-L	839	Q-E	720	P-S	174	I-V	157
T-S	455	V-A	910	S-A	769	V-A	467	T-N	257
A-S	465	S-T	912	S-A	786	N-T	509	N-T	509
N-T	485	S-D	922	S-A	827	T-S	510	T-S	510
S-P	501	A-S	927	S-L	839	H-Y	525	H-Y	525
N-H	509	A-S	942	V-A	910	S-T	578	S-A	542
T-N	510	S-T	985	S-T	912	V-G	608	S-T	578
H-Y	525	I-L	1003	S-D	922	E-D	1362	D-G	608
A-S	527	G-T	1026	A-S	927			S-A	769
A-I	528	E-N	1030	A-S	942			N-K	785
D-N	531	A-S	1044	S-T	985			E-D	805
T-A	535	N-K	1055	I-L	1003			A-T	828
K-T	542	S-A	1069	E-N	1030			N-K	888
A-T	543	S-N	1160	A-S	1044			L-V	903
I-V	558	V-I	1191	N-K	1055			K-R	909
K-N	568	I-T	1235	S-A	1069			Q-H	1254
S-T	578	T-N	1237					D-N	1260
A-G	583	F-L	1316						
F-L	604	G-S	1318						

**Table 5:** Comparison of amino acid variant sites of HVR between strain NN190313 and goat-derived bovine coronavirus strain XJCJ2301G.

NN190313						XJCJ2301G			
**aa Changes**	HVR Location	S Gene Location	aa Changes	HVR Location	S Gene Location	aa Changes	HVR Location	aa Changes	HVR Location
T-S	14	455	A-I	87	528	T-S	14	D-N	90
A-S	17	458	D-N	90	531	S-V	17	T-A	94
A-S	24	465	T-A	94	535	A-S	24	K-T	101
N-T	44	485	K-T	101	542	N-T	44	A-T	102
S-P	61	501	A-T	102	543	N-H	68	I-V	117
N-H	68	509	P-T	109	550	S-N	69	K-N	127
S-N	69	510	I-V	117	558	H-Y	84	A-G	142
H-Y	84	524	K-N	127	568	A-S	86		
A-S	86	527	A-G	142	583	A-I	87		

**Table 6:** S gene selection pressure analysis of positive selection pressure loci.

AA	FUBAR (Post Pro)	SLAC (*p*-Value)	MEME (*p*-Value)	FEL (*p*-Value)
12	+	+	+	+
113	+	+	+	+
447	+	+	+	-
458	-	+	+	+
499	+	+	+	-
501	+	+	+	+
509	+	+	+	+
510	+	+	+	-
770	+	+	+	-
1238	+	+	+	-
1363	+	+	-	+

## Data Availability

The datasets used and/or analysed during the current study are available from the corresponding author on reasonable request.
